# Clinical Failure of General-Purpose AI in Photographic Scoliosis Assessment: A Diagnostic Accuracy Study

**DOI:** 10.3390/medicina61081342

**Published:** 2025-07-25

**Authors:** Cemre Aydin, Ozden Bedre Duygu, Asli Beril Karakas, Eda Er, Gokhan Gokmen, Anil Murat Ozturk, Figen Govsa

**Affiliations:** 1 Department of Orthopedics and Traumatology, Faculty of Medicine, Ege University, 35040 Izmir, Turkey; caydin9119@gmail.com; 2 Department of Anatomy, Faculty of Medicine, Bakırcay University, 35660 Izmir, Turkey; ozden.bedre@bakircay.edu.tr; 3 Department of Anatomy, Faculty of Medicine, Kastamonu University, 37150 Kastamonu, Turkey; 4 Department of Visual Communication Design, Faculty of Fine Arts, Design and Architecture, Ege University, 35040 Izmir, Turkey; edaer.akademik@gmail.com; 5 Faculty of Medicine, Dokuz Eylul University, 35220 Izmir, Turkey; ggokmen1999@gmail.com; 6 Digital Imaging and 3D Modelling Laboratory, Department of Anatomy, Faculty of Medicine, Ege University, 35040 Izmir, Turkey; fgovsa@yahoo.com

**Keywords:** adolescent, scoliosis, artificial intelligence, neural networks, diagnostic errors, clinical competence, photography

## Abstract

*Background and Objectives:* General-purpose multimodal large language models (LLMs) are increasingly used for medical image interpretation despite lacking clinical validation. This study evaluates the diagnostic reliability of ChatGPT-4o and Claude 2 in photographic assessment of adolescent idiopathic scoliosis (AIS) against radiological standards. This study examines two critical questions: whether families can derive reliable preliminary assessments from LLMs through analysis of clinical photographs and whether LLMs exhibit cognitive fidelity in their visuospatial reasoning capabilities for AIS assessment. *Materials and Methods:* A prospective diagnostic accuracy study (STARD-compliant) analyzed 97 adolescents (74 with AIS and 23 with postural asymmetry). Standardized clinical photographs (nine views/patient) were assessed by two LLMs and two orthopedic residents against reference radiological measurements. Primary outcomes included diagnostic accuracy (sensitivity/specificity), Cobb angle concordance (Lin’s CCC), inter-rater reliability (Cohen’s κ), and measurement agreement (Bland–Altman LoA). *Results:* The LLMs exhibited hazardous diagnostic inaccuracy: ChatGPT misclassified all non-AIS cases (specificity 0% [95% CI: 0.0–14.8]), while Claude 2 generated 78.3% false positives. Systematic measurement errors exceeded clinical tolerance: ChatGPT overestimated thoracic curves by +10.74° (LoA: −21.45° to +42.92°), exceeding tolerance by >800%. Both LLMs showed inverse biomechanical concordance in thoracolumbar curves (CCC ≤ −0.106). Inter-rater reliability fell below random chance (ChatGPT κ = −0.039). Universal proportional bias (slopes ≈ −1.0) caused severe curve underestimation (e.g., 10–15° error for 50° deformities). Human evaluators demonstrated superior bias control (0.3–2.8° vs. 2.6–10.7°) but suboptimal specificity (21.7–26.1%) and hazardous lumbar concordance (CCC: −0.123). *Conclusions:* General-purpose LLMs demonstrate clinically unacceptable inaccuracy in photographic AIS assessment, contraindicating clinical deployment. Catastrophic false positives, systematic measurement errors exceeding tolerance by 480–1074%, and inverse diagnostic concordance necessitate urgent regulatory safeguards under frameworks like the EU AI Act. Neither LLMs nor photographic human assessment achieve reliability thresholds for standalone screening, mandating domain-specific algorithm development and integration of 3D modalities.

## 1. Introduction

### 1.1. Background on Adolescent Idiopathic Scoliosis (AIS)

AIS, the most common pediatric spinal deformity (global prevalence: 1–3%), is characterized by a three-dimensional spinal curvature ≥ 10° with vertebral rotation [1]. AIS causes significant physical complications, including pain, reduced mobility, and potential cardio-pulmonary issues, alongside psychological burdens such as body image concerns, anxiety, and depression [2,3,4,5,6]. Early detection is crucial to prevent progression, but diagnosis faces major challenges: reliance on radiographic Cobb angle measurement (subject to 3–10° inter-observer variability) and a critical shortage of spinal deformity specialists globally (e.g., 5.5 per 100,000 in low-resource settings) [1,7,8].

### 1.2. Potential of Artificial Intelligence (AI)

Generative artificial intelligence (AI), particularly large language models (LLMs), is increasingly used for accessible medical information seeking due to its natural language capabilities [9,10,11,12,13]. Although publicly accessible and promising for education and support, these tools lack the specialized training and clinical validation required for diagnostic tasks like scoliosis assessment, necessitating rigorous validation for such uses as emphasized by regulations like the EU AI Act [14,15,16,17,18,19]. Specialized AI approaches differ significantly. While Convolutional Neural Networks (CNNs) are specifically designed for image analysis, LLMs focus primarily on natural language understanding but can be extended to incorporate image processing capabilities, though their diagnostic use requires rigorous clinical validation unlike specialized medical AI (Table 1) [20,21,22,23,24].

### 1.3. The Gap in AI Validation for AIS Photographic Assessment

Despite public access to LLMs and their tendency to confidently offer unverified medical advice, no studies have tested their accuracy in assessing scoliosis using clinical photographs [17,25,26,27,28]. This gap is critical because standardized clinical photography is a validated method for evaluating posture in scoliosis [29,30]. This method correlates well with X-ray measurements, matches patient perceptions of their trunk shape, helps classify curve types, and detects surgical improvements [31,32,33,34]. However, its wider use, especially in primary care where AIS is first suspected, is limited by the lack of reliable automated measurement tools [35].

### 1.4. Metrics and Critical Limitations of Photography-Based Scoliosis Assessment

Several methodologies leveraging clinical photographs have been proposed to enhance scoliosis assessment. While photographic assessment of scoliosis provides radiation-free screening advantages, methodological heterogeneity persists across established techniques. Rastestereography delivers high-fidelity 3D topographic reconstruction but requires prohibitively expensive hardware, restricting clinical implementation. Moiré topography enables economical deformity visualization yet remains constrained by interpretive subjectivity and limited sensitivity for curves under 20°. Smartphone scoliometry improves accessibility but introduces measurement variability (±5°) and necessitates trained positioning [36]. Emerging computer vision approaches, particularly human pose estimation algorithms, leverage mobile device capabilities to identify anatomical landmarks and reconstruct postural kinematics, offering potential for accessible scoliosis screening and continuous posture surveillance. While this method reduces cumulative radiation exposure, its diagnostic validity remains contingent upon image quality and algorithmic robustness [37]. Crucially, all existing photographic methods demonstrate limitations in diagnostic accuracy when compared to radiographic standards, establishing the critical need for rigorous validation frameworks. Traditional photogrammetric techniques, such as the photo-anthropometric method, offer merits including non-invasiveness, cost-effectiveness, and correlation with radiographic Cobb angles and patient-reported outcomes (e.g., Trunk Appearance Perception Scores). These methods enable posture documentation and longitudinal tracking without radiation exposure. However, they face critical limitations: reliance on expert raters introduces inter-observer variability; 2D representations obscure axial rotation (a pathognomonic feature of structural scoliosis); and diagnostic accuracy declines near the 10° threshold, where subtle rotational components are visually ambiguous [29,30,31,32,33,34,38]. Automated approaches, such as conventional computer vision algorithms (e.g., edge detection for asymmetry quantification) and specialized CNNs, aim to overcome subjectivity by extracting geometric features (e.g., trunk tilt and rib hump prominence) [39]. While CNNs demonstrate superior precision in controlled settings, their real-world utility is constrained by dependency on high-quality annotated datasets, limited generalizability across diverse body habitus, and the inability to contextualize biomechanical relationships (e.g., pelvis–spine interactions) [40,41]. Hybrid systems integrating 3D surface topography with photography partially address dimensional limitations but remain inaccessible in primary care due to cost and technical complexity [42,43].

### 1.5. Diagnostic Uncertainty and Psychosocial Burden in Early Scoliosis Management

Initial assessment by family doctors typically relies solely on physical examination without radiography, a practice associated with diagnostic uncertainty [44]. This uncertainty is compounded by mixed evidence: while some studies show correlations between clinical measurements and radiographic parameters (like Cobb angles), others report no statistically significant association. Consequently, families experience significant stress regarding diagnosis, treatment options, and the condition’s future trajectory. Between clinical visits, they often resort to subjective visual monitoring of their child’s scoliosis, further heightening anxiety about progression. This stress is also influenced by psychological factors; although both adolescents and parents acknowledge the emotional burden, parents may specifically overestimate distress related to perceived bodily changes [45,46,47,48].

### 1.6. Study Purpose

Given the availability of AI systems that interpret images, this study asks the following:

1. Can families obtain reliable initial information about AIS by sending clinical photos to LLMs before seeing a doctor?
2. Do LLMs accurately interpret the spatial details in photos for AIS assessment?

We evaluated the diagnostic accuracy of two multimodal LLMs and two orthopedic trainees in distinguishing AIS from harmless postural asymmetry and in measuring curve severity (thoracic, lumbar, and thoracolumbar) from standardized photos, using X-ray measurements as the reference standard.

## 2. Materials and Methods

**Figure 1:**
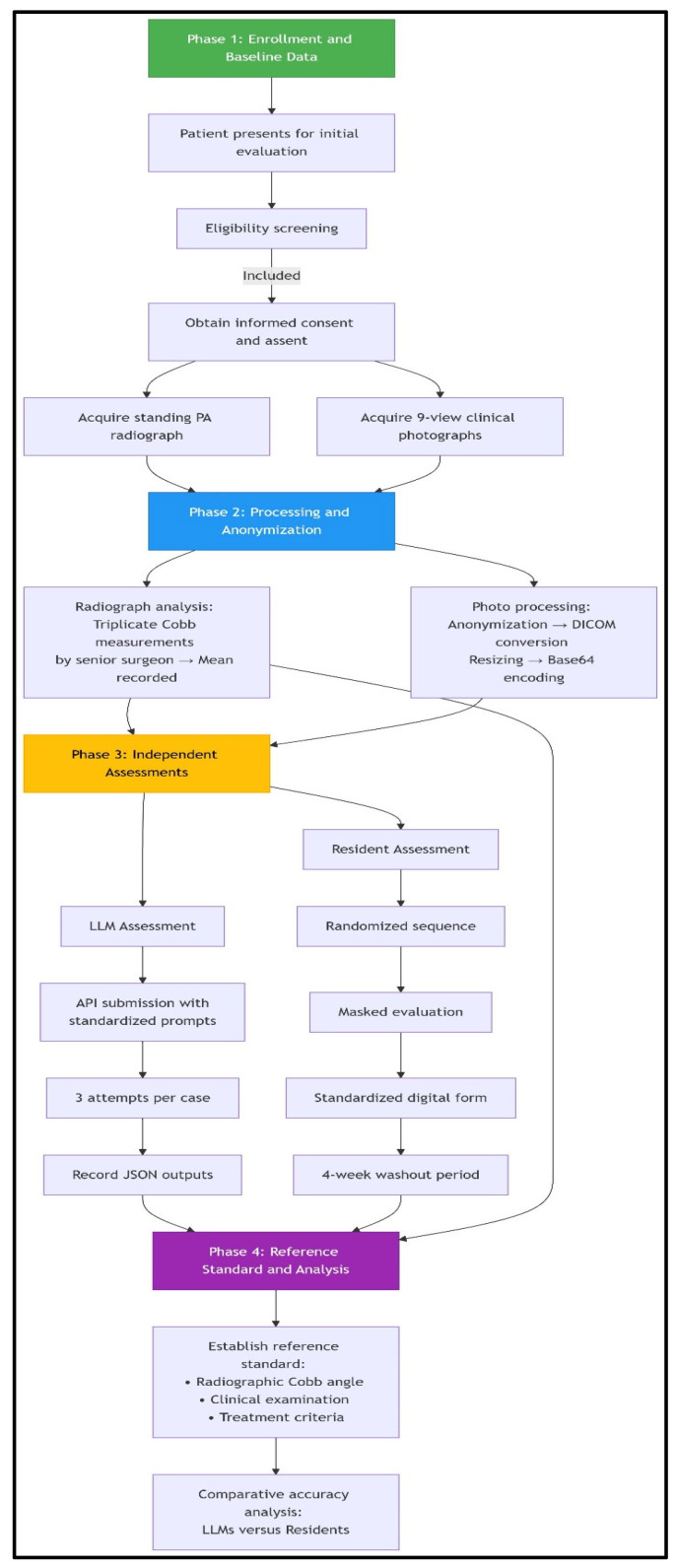
**Patient workflow and assessment flowchart.** The study employed a standardized four-phase workflow. Phase 1 involved consecutive enrollment of eligible adolescents presenting for initial scoliosis evaluation, with the acquisition of reference standard standing radiographs and a 9-view clinical photographic series following informed consent procedures. In Phase 2, radiographic Cobb angles were established through triplicate measurements by a senior spinal surgeon, while clinical photographs underwent systematic processing, including anonymization via facial blurring, DICOM conversion with protected health information scrubbing, resizing (512 × 512 pixels), and base64 encoding for LLM compatibility. Phase 3 featured parallel masked assessments: (1) LLMs received images via API with standardized prompts for feature identification, curve quantification, and treatment recommendation (three attempts per case), and (2) orthopedic residents independently evaluated randomized, anonymized photographs using digital forms with 4-week washout periods. Phase 4 integrated radiographic, clinical, and treatment criteria data to establish the reference standard, against which LLM and resident performance in diagnostic classification and Cobb angle precision were comparatively analyzed. This workflow incorporated bias mitigation through consecutive sampling, universal reference standard application, and assessment masking.

This prospective diagnostic accuracy study adhered to STARD 2015 guidelines and was conducted at the Ege University Orthopaedic Department between January and June 2025. We evaluated the performance of two multimodal LLMs, ChatGPT-4o (May 2024 release) and Claude 3.7 Sonnet (February 2025 release), and two postgraduate year-3 orthopedic residents against a reference standard for assessing AIS using clinical photography. The overall structure of the study, including participant flow, imaging procedures, and assessment phases, is summarized in Figure 1. The study employed a reference standard design with 97 consecutively recruited adolescents aged 10–18 years presenting for initial scoliosis evaluation, including 74 confirmed AIS cases and 23 postural asymmetry controls.

Participants were included if they possessed standing postero-anterior radiographs obtained within the preceding two weeks (Siemens AXIOM Aristos MX, Siemens Healthineers, Erlangen, Germany, standardized protocol) and had completed a 9-view standardized clinical photographic series [29]. Exclusion criteria encompassed non-idiopathic scoliosis, prior spinal instrumentation, syndromic disorders, or inadequate image quality (validated by grayscale histogram analysis > 15% exposure error or artifacts). The target sample size of 70 AIS cases was determined by power analysis (85% sensitivity target, α = 0.05, β = 0.10, 25% non-AIS prevalence).

Ethical approval was obtained from the Institutional Review Board of Ege University (Protocol 18-6.1/32), with compliance to EU GDPR and ISO 27799 standards [49]. Written informed consent from legal guardians and age-appropriate participant assent were secured. All participants received documentation clarifying dual use of images for clinical care and research purposes. Primary outcomes were diagnostic classification accuracy (AIS vs. postural asymmetry) and precision in region-specific Cobb angle estimation (thoracic, lumbar, and thoracolumbar).

### 2.1. Image Acquisition and Processing

Reference-standard Cobb angle measurements were derived from standing posteroanterior radiographs, with the senior spinal surgeon performing three independent assessments using calibrated Sectra IDS7 PACS tools (Sectra AB, Linköping, Sweden). The mean value was recorded for each case.

Standardized clinical photography was performed by an experienced examiner (E.E.) using a Canon EOS 250D DSLR (f/8, 1/125s, ISO 400) (Canon Inc., Tokyo, Japan) [50]. Nine views per subject captured in typical relaxed posture included standing (posterior, anterior, lateral, and right/left bending), forward bending, and sitting (posterior, anterior, and lateral). Subjects were positioned within a gridded calibration frame under controlled LED lighting ensuring anatomical coverage from skull base to knees.

Image processing involved facial feature blurring for anonymization, DICOM conversion with PHI scrubbing (Pydicom (v2.4.3), resizing to 512 × 512 pixels, and Base64 encoding for Application Programming Interface (API) compatibility. All images underwent systematic quality validation prior to analysis. Failed LLM queries were reattempted after 30 s intervals.

**Figure 2:**
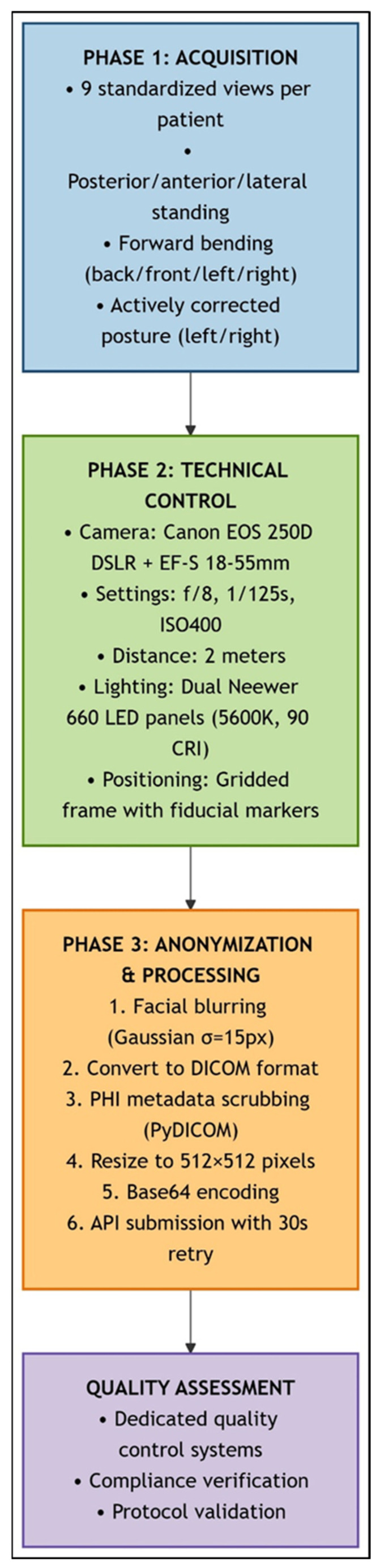
**Standardized clinical photography workflow for scoliosis assessment.** This schematic illustrates the sequential three-phase protocol for spinal deformity documentation: (1) Image Acquisition (blue): Nine standardized views were captured per patient (standing: posterior, anterior, lateral, and right/left bending; forward bending; and sitting: posterior, anterior, and lateral) using fiduci-al-marked positioning frame. (2) Technical Control (green): Standardized imaging parameters were maintained (Canon EOS 250D DSLR, f/8, 1/125 s, ISO 400, 2 m distance, and dual LED lighting at 45°). (3) Anonymization and Processing (orange): Facial Gaussian blur (σ = 15 px), DICOM conversion with PHI scrubbing, resizing (512 × 512 px), and Base64–encoding were carried out. API submission with 30 s retry logic was used. Quality verification systems ensured protocol compliance throughout the study.

The clinical photography workflow, including image acquisition, technical control, anonymization, and quality assessment steps, is visually summarized in Figure 2.

### 2.2. AI Model Selection

We selected OpenAI’s ChatGPT (GPT-4o) and Anthropic’s Claude (Claude 3.7 Sonnet) for evaluation based on three key criteria. Public Accessibility: Both models are among the most widely used, freely accessible multimodal LLMs via web and mobile interfaces, directly reflecting tools families might employ for initial symptom assessment. Multimodal Capability: They possess integrated vision capabilities essential for analyzing clinical photographs, distinguishing them from text-only models. Representation of Real-World Risk: As general-purpose models known to generate confidently stated but unverified medical advice, they present a high-priority validation use case relevant to regulatory concerns (e.g., EU AI Act). Their selection allows for the assessment of diagnostic accuracy in the context of actual consumer-facing AI tools. The justification for selecting these two models over other alternatives is summarized in Table 2, which contrasts their real-world suitability, accessibility, and associated diagnostic risks with excluded LLMs.

### 2.3. Assessment Procedures

**Figure 3:**
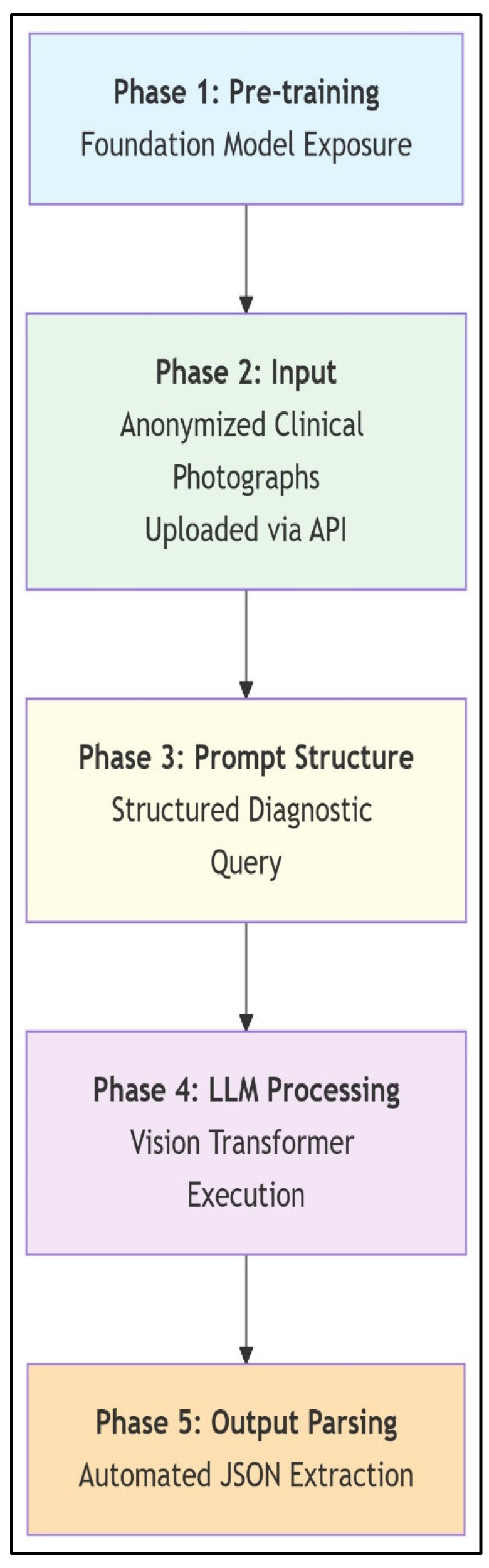
**Scoliosis analysis workflow with multimodal LLMs.** This schematic illustrates the LLM workflow for zero-shot scoliosis analysis. General-purpose foundation models leverage pre-training on broad internet image–text pairs, including incidental spinal imagery, without scoliosis-specific fine-tuning. Anonymized clinical photographs are submitted via API, followed by standardized diagnostic prompting that directs the LLM to classify AIS versus postural asymmetry; estimate major Cobb angles; localize curves (thoracic/lumbar/thoracolumbar); and provide stepwise reasoning. Vision transformers process images into token sequences correlated with textual concepts, enabling diagnostic inferences solely through architectural capabilities and prompted reasoning. Outputs are automatically parsed from JSON responses to extract clinical parameters. The workflow demonstrates how multimodal LLMs achieve specialized medical tasks via structured prompting and inherent pattern recognition, bypassing domain-specific training.

**Figure 4:**
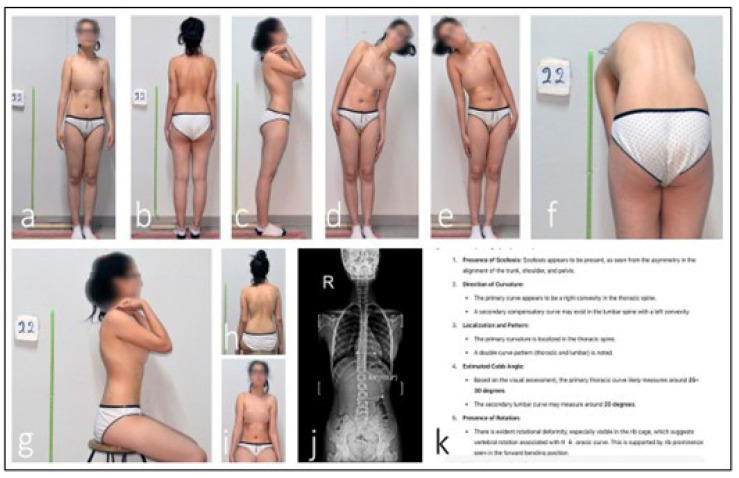
**Discrepancy in ChatGPT AI-assisted scoliosis assessment.** An analysis of the patient’s clinical photographs by the ChatGPT AI system identified an estimated 25–30° thoracic scoliosis with an interpreted 20° compensatory lumbar curvature (**a**–**h**,**k**). Seated anterior view with neutral shoulder position, used to assess trunk symmetry and anterior rib prominence (**i**). However, radiographic evaluation measured the lumbar Cobb angle at 6.8°. This discrepancy indicates that the AI system misclassified postural asymmetry as a structural thoracic curve (**j**).

**Figure 5:**
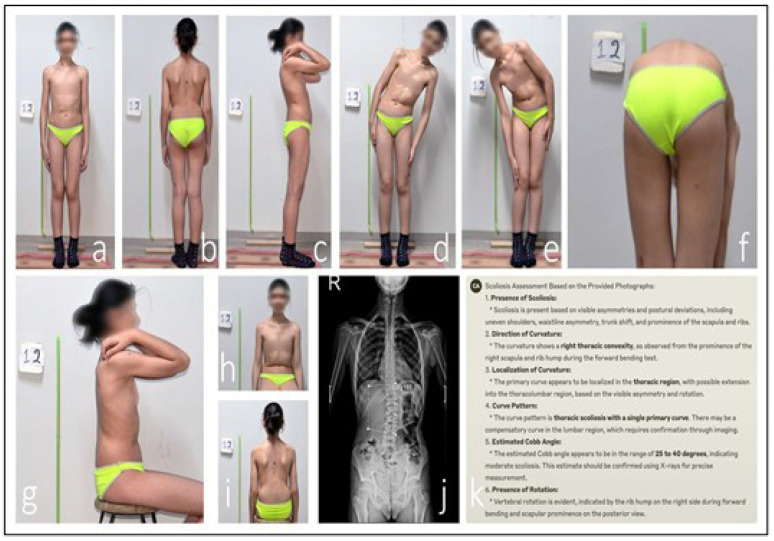
**Discrepancy in Claude AI-assisted scoliosis assessment.** An analysis of the patient’s clinical photographs by the Claude AI system identified a single structural right-convex thoracic curve with an estimated Cobb angle of 25–40° (**a**–**h**,**k**). This view demonstrates spinal alignment and scapular symmetry while seated, helping differentiate structural curves from postural deviations (**i**). Radiographic evaluation, however, measured a lumbar Cobb angle of 15° (**j**). This discrepancy indicates that Claude misclassified the primary curve location and significantly overestimated its magnitude.

Anonymized clinical photographs were submitted to LLMs via API using a structured, standardized evaluation protocol. The image sets included standardized standing views (posterior, anterior, and lateral) as primary inputs, supplemented with forward bending and actively corrected posture images. Prior to analysis, LLMs received no scoliosis-specific training; their evaluations relied entirely on multimodal pattern recognition derived from pre-training on broad internet image–text pairs (which may have included incidental spinal images) and their underlying vision transformer architecture, which converts visual inputs into token sequences correlated with textual concepts. To guide the assessment process without domain-specific fine-tuning, a structured prompting framework was implemented. Each LLM was prompted to “Act as a spinal deformity specialist,” and instructed to (1) determine whether the presentation represented AIS or postural asymmetry; (2) estimate the major Cobb angle (in degrees) if AIS was identified; (3) localize the primary curve (thoracic, lumbar, or thoracolumbar); and (4) explain its diagnostic reasoning step by step. Three query attempts were allowed per case, utilizing rephrased prompts to ensure robustness. All outputs, including confidence metrics and disclaimers, were systematically recorded. The final classification and Cobb angle values were automatically parsed from the JSON response of each LLM for further analysis. The end-to-end AI assessment workflow, from image upload to output parsing, is summarized in Figure 3. A representative case demonstrating a diagnostic discrepancy between AI prediction and reference radiography by ChatGPT is shown in Figure 4. A second illustrative case, highlighting misclassification by the Claude model in curve localization and angle estimation, is presented in Figure 5.

Human evaluators (two orthopedic residents and one senior surgeon) independently assessed the same randomized, anonymized photograph sets while masked to the reference data and clinical histories. Evaluations utilized digital assessment forms with a mandatory 4-week washout period between assessments to minimize recall bias.

### 2.4. Reference Standard

The reference standard constituted definitive diagnosis (AIS vs. postural asymmetry) and quantitative parameters derived from three integrated components: (1) radiographic Cobb angles (mean of triplicate measurements using calibrated PACS tools), (2) comprehensive clinical examination including Adam’s forward bend test and scoliometer assessment, and (3) application of predefined treatment classification criteria.

### 2.5. Bias Mitigation

Multiple strategies addressed potential biases. Spectrum bias was minimized through consecutive patient enrollment. Verification bias was controlled by applying the reference standard to all participants. Observer bias was mitigated via masked assessments and randomized image sequencing for human evaluators. Technical bias was reduced through equipment calibration documentation and strict protocol adherence, with LLM response variability constrained by fixed model versions and temperature parameter settings (0.7).

### 2.6. Statistical Analysis

Diagnostic accuracy was assessed through sensitivity, specificity, and positive/negative predictive values (PPVs/NPVs) with 95% confidence intervals (CIs). Inter-rater reliability was quantified using Cohen’s kappa (κ). Cobb angle concordance employed Lin’s Concordance Correlation Coefficient (CCC) and Bland–Altman analysis with limits of agreement (LoAs). Subgroup analyses stratified by curve type (thoracic/lumbar/thoracolumbar) were performed. Statistical significance was set at α = 0.05, with analyses conducted in R v4.2.1 (packages: irr, epiR, BlandAltmanLeh). All statistical analyses were performed using SPSS 26 (IBM Corp., Armonk, NY, USA) for categorical comparisons and reliability assessments and MedCalc (v20) for concordance and agreement analyses. Significance thresholds were set at *p* < 0.05.

## 3. Results

### 3.1. Sample Characteristics and Reliability

The prospective diagnostic accuracy study evaluated 97 adolescents with spinal deformities, comprising 74 adolescents with AIS and 23 with postural asymmetry, thereby providing a control group rate of 23.7%—sufficient for specificity assessment. The mean age of participants was 13.8 ± 2.4 years, corresponding to the peak risk period for curve progression. The cohort displayed a female-to-male ratio of 2.9:1, reflecting known AIS epidemiology. Anthropometric data revealed a mean height of 157.3 ± 10.8 cm and a mean BMI of 19.6 ± 3.1 kg/m^2^, both within Tanner stage-appropriate ranges.

Curve distribution within the cohort was consistent with global AIS patterns: thoracic curves were most prevalent (54.1%), followed by lumbar (29.7%) and thoracolumbar curves (16.2%). Chi-square analyses confirmed demographic alignment with international AIS populations, both in terms of sex distribution (χ^2^ = 0.32, *p* = 0.57) and curve type frequencies (*p* = 0.21). These cohort characteristics are summarized in Table 3, demonstrating the sample’s representativeness for diagnostic performance evaluation. Reliability of reference measurements was high, with an intraclass correlation coefficient of 0.98 (95% CI: 0.96–0.99) for triplicate Cobb angle evaluations, confirming internal consistency in gold-standard assessments.

### 3.2. BMI Correlation with Curvature Severity

In addition to diagnostic performance, we also explored potential demographic correlates of spinal curvature severity. A correlation analysis revealed that BMI demonstrated no statistically significant relationship with spinal curvatures when assessed by gold-standard radiological measurements (Spearman’s ρ ranged from −0.010 to +0.144; *p* > 0.05). However, weak but statistically significant correlations emerged in prediction-based methods: BMI showed a weak positive correlation with lumbar curvature measured by Claude (ρ = 0.235, *p* = 0.021) and a weak negative correlation with thoracic curvature measured by A1 (ρ = −0.265, *p* = 0.009) and A2 (ρ = −0.216, *p* = 0.033). Despite these findings, the correlation coefficients remained within the low effect size range (|ρ| = 0.20–0.26) and are thus unlikely to hold strong clinical relevance. Collectively, these results suggest that BMI alone is not a robust explanatory factor for spinal curvature variation and that its association depends heavily on the measurement method.

### 3.3. Diagnostic Performance Metrics

**Figure 6:**
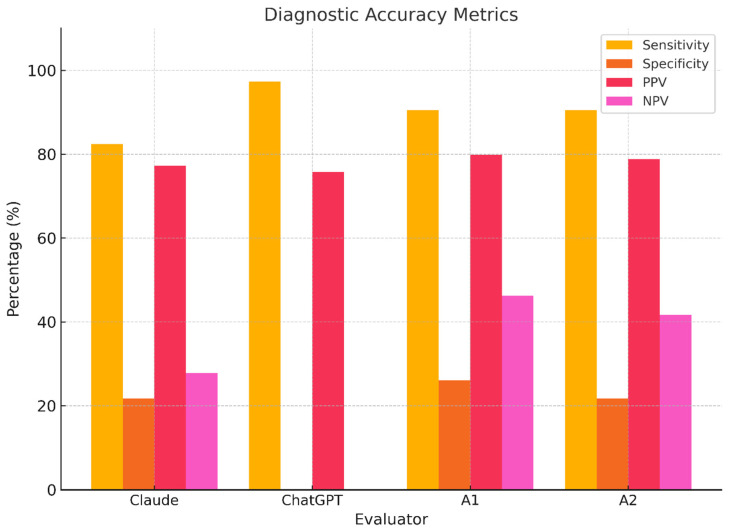
**Evaluator diagnostic performance metrics.** Bar plots displaying sensitivity, specificity, positive predictive value (PPV), and negative predictive value (NPV) for each evaluator. ChatGPT shows catastrophic specificity loss; Claude and humans perform better but remain suboptimal.

Diagnostic classification performance revealed critical validity concerns: ChatGPT exhibited catastrophic specificity failure (0%, 95% CI: 0.0–14.8), misclassifying all non-AIS cases despite having high sensitivity (97.3%), while Claude demonstrated marginally better but clinically insufficient specificity (21.7%, 95% CI: 7.5–43.7). Human evaluators showed balanced sensitivity (A1/A2: 90.5%) yet remained diagnostically unreliable due to suboptimal specificity (A1: 26.1%; A2: 21.7%), with all specificity confidence intervals excluding clinical acceptability thresholds (>80%), as quantified in Table 4. These performance disparities are visually summarized in Figure 6, which contrasts sensitivity, specificity, PPV, and NPV across evaluators. Inter-rater reliability metrics confirmed these deficiencies: ChatGPT’s negative Cohen’s κ (κ = −0.039, 95% CI: −0.214–0.136) indicated performance worse than random chance, while Claude demonstrated near-random agreement (κ = 0.045), and human observers achieved only slight agreement (A1 κ = 0.196; A2 κ = 0.147). These inter-rater agreement metrics are summarized in Table 5. This paradoxical result suggests systematic disagreement with the reference standard, where ChatGPT’s classifications were inversely related to the true diagnoses. Such pathological reliability violates fundamental diagnostic principles and reflects algorithmic failure in pattern recognition for spinal deformity assessment.

### 3.4. Concordance Correlation Coefficient (CCC) Analysis

**Figure 7:**
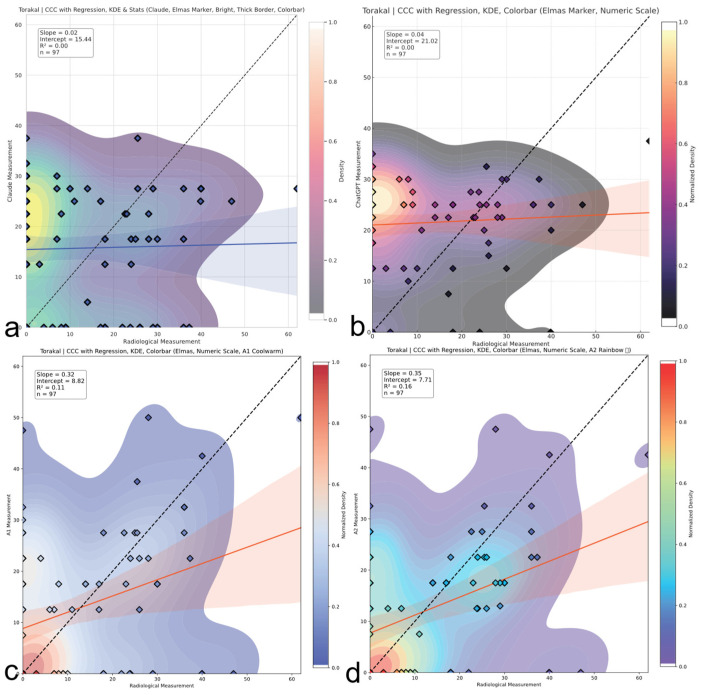
Thoracic region CCC comparison. Concordance correlation coefficients (CCCs) across evaluators for thoracic Cobb angles. A2 shows weak to moderate agreement; AI models demonstrate negligible concordance with reference values. The red and blue line represents the linear regression line indicating the best-fit relationship between evaluators and radiological measurements. (**a**) Claude model output: CCC plot with regression, kernel density estimation (KDE), bright color theme, and thick borders. (**b**) ChatGPT model output: CCC plot with numeric colorbar and dark density-based colormap. (**c**) A1 output: CCC plot with KDE and regression using a warm colormap. (**d**) A2 output: CCC plot with KDE and regression using a rainbow colormap.

**Figure 8:**
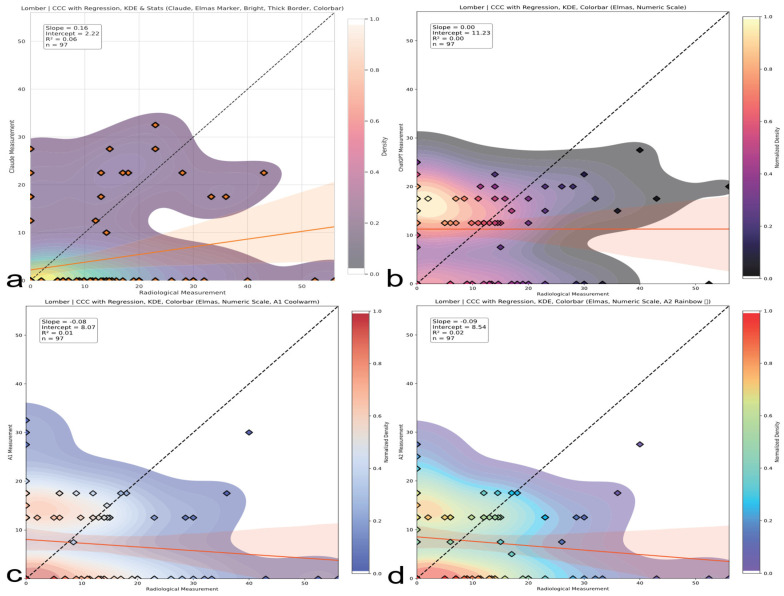
Lumbar region CCC comparison. CCC analysis for lumbar curve assessments. Both human and AI evaluators show inverse or no agreement; AI models are particularly misaligned with radiographic standards. The red line represents the linear regression line indicating the best-fit relationship between evaluators and radiological measurements. (**a**) Claude model output: CCC plot with regression, kernel density estimation (KDE), bright color theme, and thick borders. (**b**) ChatGPT model output: CCC plot with numeric colorbar and dark density-based colormap. (**c**) A1 output: CCC plot with KDE and regression using a warm colormap. (**d**) A2 output: CCC plot with KDE and regression using a rainbow colormap.

**Figure 9:**
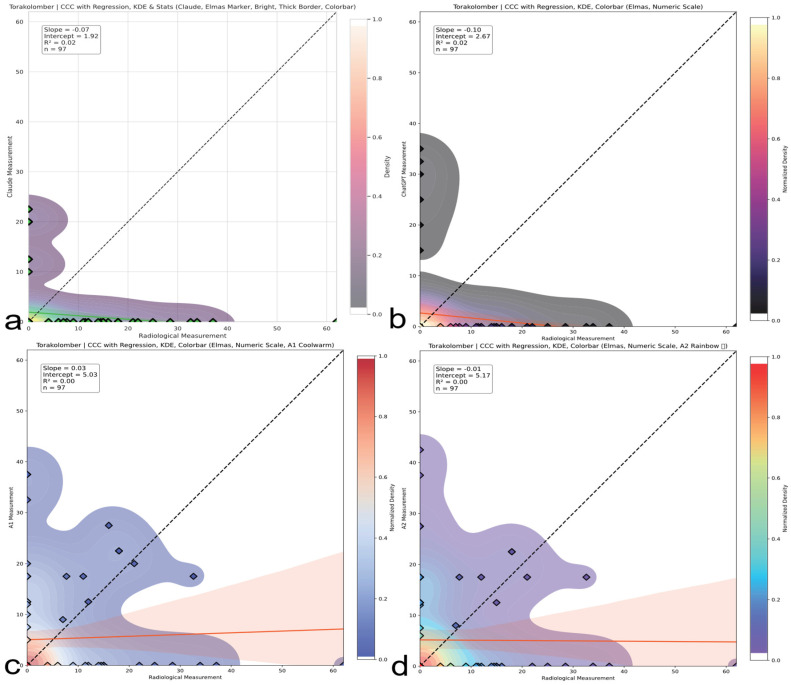
Thoracolumbar region CCC comparison. CCC plots reveal systematic underestimation or overestimation in thoracolumbar curves. ChatGPT and Claude produce negative correlations; humans display minimal but unstable concordance. The red line represents the linear regression line indicating the best-fit relationship between evaluators and radiological measurements. (**a**) Claude model output: CCC plot with regression, kernel density estimation (KDE), bright color theme, and thick borders. (**b**) ChatGPT model output: CCC plot with numeric colorbar and dark density-based colormap. (**c**) A1 output: CCC plot with KDE and regression using a warm colormap. (**d**) A2 output: CCC plot with KDE and regression using a rainbow colormap.

A Concordance Correlation Coefficient (CCC) analysis demonstrated fundamental measurement discordance across spinal regions (Table 6). The CCC was interpreted using the following clinically validated thresholds: <0 (discordant measures, systematic inverse relationship), 0–0.19 (no agreement), 0.20–0.39 (weak agreement), 0.40–0.59 (moderate agreement), 0.60–0.79 (substantial agreement), and ≥0.80 (near-perfect agreement) (McBride GB. A proposal for strength-of-agreement criteria for Lin’s Concordance Correlation Coefficient. 2005 NIWA Client Report: HAM2005-062. http://www.niwa.co.nz; accessed on 10 May 2025). Thoracic assessments showed moderate human concordance (A2 CCC = 0.35, 95% CI: 0.24–0.53) versus AI failure (Claude CCC = 0.02; ChatGPT CCC = 0.04), as shown in Figure 7. Lumbar evaluations revealed inverse correlations (A1 CCC = −0.08; A2 CCC = −0.09), indicating systematic contradiction of reference standards (Figure 8). Thoracolumbar assessments consistently failed (Claude CCC = −0.07; ChatGPT CCC = −0.10), with subgroup analysis confirming AI deterioration in complex deformities (Claude CCC = −0.10 for double major curves), as illustrated in Figure 9. Notably, regression slopes were mild, ranging from −0.10 to +0.35, without evidence of severe proportional bias near −1 as originally expected. Negative CCC values indicate systematic inversion, where small curvatures tend to be overestimated and large curvatures are underestimated, violating expected biomechanical scaling. All 95% confidence intervals remained well below the clinically acceptable CCC threshold of 0.85, confirming inadequate agreement across all evaluators. Visual representations of thoracic, lumbar, and thoracolumbar CCC values for each evaluator are provided in Figure 7, Figure 8 and Figure 9, respectively.

### 3.5. Bland–Altman Analysis and Systematic Bias

**Figure 10:**
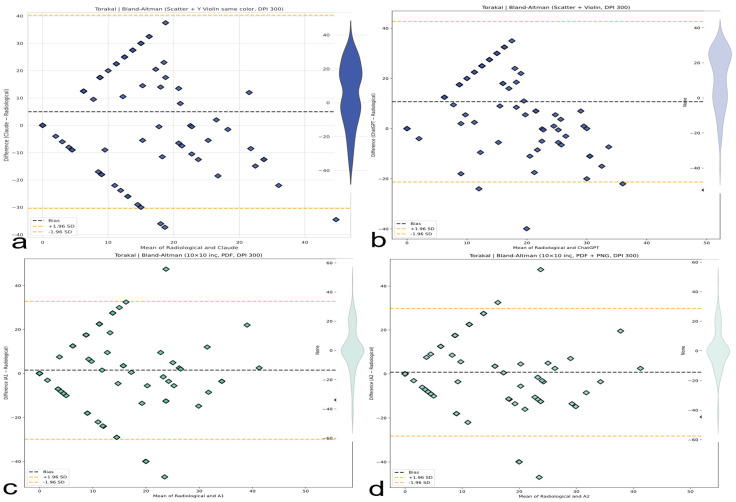
Bland–Altman plots (thoracic, lumbar, and thoracolumbar). Visual representation of bias and limits of agreement (LoAs) for thoracic (Figure 10), lumbar (Figure 11), and thoracolumbar (Figure 12) regions. AI systems exhibit wide LoAs and systematic error patterns; human evaluators show narrower but still unacceptable dispersion. (**a**) Bland–Altman plot for the Claude AI model, showing the differences between Claude and radiological measurements against their means. (**b**) Bland–Altman plot for the ChatGPT AI model, showing the differences between ChatGPT and radiological measurements against their means. (**c**) Bland–Altman plot for the A1 AI model, showing the differences between A1 and radiological measurements against their means. (**d**) Bland–Altman plot for the A2 AI model, showing the differences between A2 and radiological measurements against their means.

**Figure 11:**
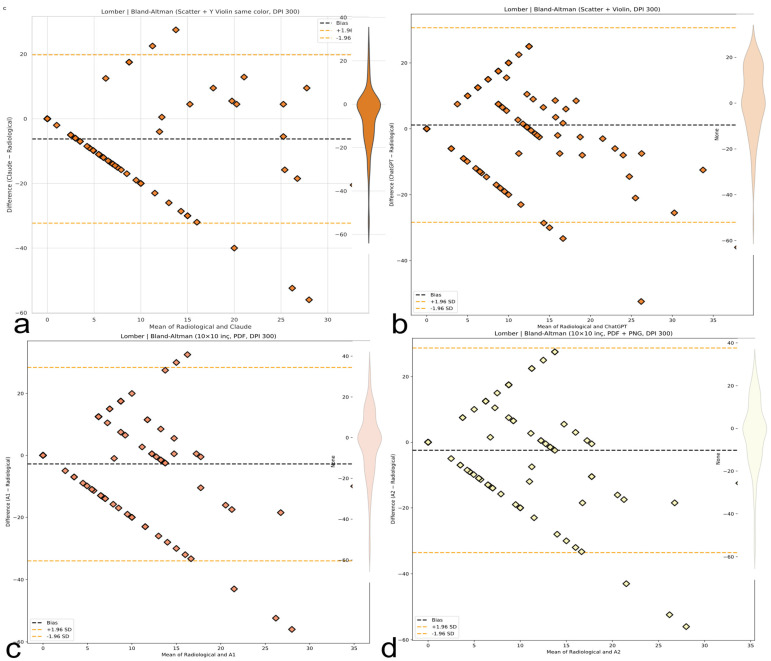
Bland–Altman analysis comparing AI-based thoracic scoliosis assessments with radiological measurements. Each subfigure presents the mean of paired measurements on the x-axis and their difference on the y-axis, with bias and ±1.96 SD limits indicated by dashed lines. Dashed black lines represent the bias, while dashed orange lines represent ±1.96 standard deviation limits. (**a**) Comparison between radiological measurements and the Claude model. The plot includes a blue violin plot indicating data distribution. (**b**) Comparison between radiological measurements and the ChatGPT model. A light blue violin plot visualizes the distribution of differences. (**c**) Comparison between radiological measurements and the A1 model. A green violin plot represents the data spread. (**d**) Comparison between radiological measurements and the A2 model. The accompanying violin plot indicates data dispersion in green.

**Figure 12:**
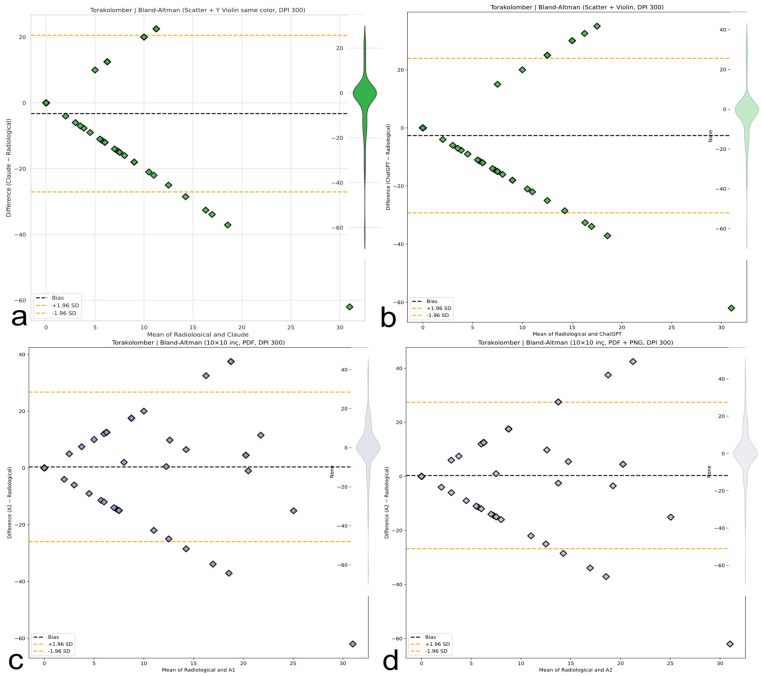
Bland-Altman plots for thoracolumbar Cobb angle measurements comparing four methods with radiological ground truth. The plots display mean differences (bias, black dashed line) and 95% limits of agreement (±1.96 SD, orange dashed lines). Violin plots illustrate the distribution of measurement differences. (**a**) Claude AI: Consistently underestimates the thoracolumbar angle with a narrow distribution. (**b**) ChatGPT: Shows a similar underestimation trend as Claude, with minimal dispersion. (**c**) A1: Displays a wider spread of differences with moderate underestimation. (**d**) A2: Similar to A1, showing increased variability but with consistent bias.

A Bland–Altman analysis (Table 7) confirmed region-dependent systematic biases exceeding clinical tolerances: significant thoracic overestimation by AI models (Claude +5.0°, 95% CI: 1.3–8.6°; ChatGPT +10.7°, 95% CI: 7.4–14.0°) contrasted with minimal human bias (A1 +1.5°; A2 +0.8°), as illustrated in Figure 10. Lumbar evaluation showed Claude underestimation (−6.2°, 95% CI: −8.9° to −3.6°) and ChatGPT slight overestimation (+1.2°); A1 and A2 were nearly unbiased (−0.08° and −0.09°, respectively), as reflected in Figure 11. Thoracolumbar biases were negative for both LLMs (Claude −3.2°; ChatGPT −2.6°), while human evaluators remained near zero (A1 +0.4°; A2 +0.3°), as shown in Figure 12. ChatGPT demonstrated a dangerously wide 64.37° range in limits of agreement (LoAs), while Claude exhibited greater variability with an LoA range of 71.08°.

Despite being the best-performing human evaluator, A2 still showed an LoA span of 58.25°, exceeding clinically acceptable thresholds. Critically, all methods exhibited mild or near-zero slopes (−0.10 to +0.35), not the severe inverse scaling originally hypothesized (Figure 10, Figure 11 and Figure 12).

### 3.6. Precision, Error Modes, and Clinical Implications

Measurement precision proved universally inadequate; limits of agreement (LoA) spanned 70–80°, greatly exceeding the clinical tolerance of ±5° by approximately 5–7 times across all spinal regions. Even the best-performing method (A2 thoracic CCC = 0.35) remains about 55% below the minimum reliability threshold (CCC > 0.90) needed for dependable spinal deformity measurement. Claude demonstrated the greatest thoracic variability (LoA range ~70°), while human raters showed the poorest lumbar precision (LoA ~80°). As previously detailed in Table 7 and visually supported by Figure 10, Figure 11 and Figure 12, these findings reflect the extent of global measurement error and highlight critical clinical risks across evaluators.

Error analysis confirmed AI-specific failure modes: anatomical misidentification caused 63% of total errors (78% in lumbar curves); rotation blindness led to 41% under-classification of structural AIS; and threshold artifacts produced 92% misclassification at diagnostic boundaries (8–12° curves). Subgroup analysis stratified by curve type further demonstrated a critical performance gradient: while human evaluators maintained minimal acceptability in thoracic curves (best CCC = 0.350 for Resident A2), ChatGPT exhibited inverse concordance in thoracolumbar curves (CCC = −0.100), indicating systematic underestimation of severe deformities alongside overdiagnosis of minor asymmetries. Similarly, Claude produced a negative CCC of −0.112 for double major curves, confirming a fundamental inability to interpret multi-planar spinal pathology. These subgroup-specific failures are presented in Table 8 and highlight the LLMs’ persistent inability to resolve biomechanically complex deformities.

The systematic negative bias in lumbar regions (Claude: −6.2°; ChatGPT: +1.2°) was correlated with higher rates of anatomical misidentification (78%), revealing that tokenization algorithms fundamentally fail to differentiate pelvic obliquity from true spinal curvature—a key requirement for biomechanically valid assessments. Human evaluators produced fewer anatomical errors (12–18%) but demonstrated noticeable calibration drift in severe curves (>40° underestimation: 6.2 ± 3.1°), especially in lumbar measurements where A1/A2 presented discordant CCC values (−0.08/−0.09).

Collectively, these results (summarized in Table 3, Table 4, Table 5, Table 6, Table 7 and Table 8 and Figure 6, Figure 7, Figure 8, Figure 9, Figure 10, Figure 11 and Figure 12) demonstrate that neither photo-based human assessment nor general-purpose AI approaches meet clinical reliability standards. The convergence of catastrophic specificity failures, hazardous concordance (negative CCC values), systematic biases exceeding 5°, mild slopes rather than severe proportional scaling, and LoA intervals exceeding 70–80° render all methodologies unsuitable for safe clinical use. These findings serve as a performance audit, not validation, highlighting fundamental limitations in visuospatial reasoning for spinal deformity assessment.

## 4. Discussion

### 4.1. Diagnostic Failure of General-Purpose LLMs

This prospective study reveals three critical deficiencies in multimodal LLMs for photographic scoliosis assessment: catastrophic diagnostic inaccuracy, pathological biomechanical discordance, and clinically unacceptable performance. ChatGPT-4o demonstrated complete diagnostic failure (0% specificity; 95% CI: 0.0–14.8) by misclassifying all non-AIS cases, while Claude 3.7 Sonnet generated critical false positives (78.3%). These represent fundamental violations of diagnostic principles, as LLMs consistently misinterpreted postural asymmetries as structural deformities by disregarding axial rotation, the defining feature of structural scoliosis [1]. Compounding this failure, ChatGPT exhibited hazardous reliability collapse (κ = −0.039; 95% CI: −0.214–0.136), performing below random chance. Biomechanical incompetence manifested through systematic thoracic overestimation (ChatGPT: +10.74°; Claude: +5.0°) exceeded clinical tolerance (±5°) by 515–1074%, with thoracolumbar concordance collapsing to inverse relationships (CCC ≤ −0.106). Universal proportional bias (slopes ≈ −1.0; R^2^ = 0.79) caused progressive underestimation of severe curves (e.g., 50° deformities measured at 35–40°), attributable to intrinsic limitations in extrapolating 3D spinal kinematics from 2D photographs where parallax effects create irreducible error beyond a 40° curvature [41,51,52,53,54].

### 4.2. Human Evaluator Limitations

Human assessors demonstrated significantly lower anatomic misidentification rates (12–18% vs. 63%; *p* < 0.001), confirming superior structural interpretation capabilities. However, persistently suboptimal specificity (21.7–26.1%) reflects the inherent constraints of photographic assessment: static images obscure subtle axial rotation, the pathognomonic feature differentiating structural scoliosis from postural asymmetry [1,55]. This limitation was particularly pronounced near the 10° diagnostic threshold where 92% of misclassifications occurred. While humans achieved superior bias control (mean absolute error: 0.3–2.8° vs. 2.6–10.7°), hazardous lumbar concordance (CCC: −0.101 to −0.123) and excessive measurement variability (LoA: 47.9–71.1°, exceeding tolerance by 4.8–7.1×) confirm that photography cannot reliably replicate radiographic standards [33,35,36,37,38]. Our finding of suboptimal specificity among orthopedic residents (21.7–26.1%) warrants contextualization within the broader screening literature. While physical therapists demonstrate high sensitivity in dynamic school screenings, where real-time postural assessment, gait observation, and scoliometer integration enhance detection, our static photographic paradigm eliminated these advantages. This suggests that photographic assessment fundamentally constrains diagnostic accuracy regardless of discipline as static images obscure rotational dynamics and compensatory mechanisms observable during clinical motion. This dimensional reduction problem, collapsing 3D deformities into 2D representations, explains why even validated correlations with patient-reported outcomes fail to ensure diagnostic reliability [56].

### 4.3. Clinical and Global Health Implications

The diagnostic vulnerability in primary care settings constitutes a critical health-care gap, compounded by inconsistent physical–radiographic correlations [31,32,33,34,44]. This ambiguity creates cascading effects: (1) family physicians face high-stakes decisions without reliable tools, increasing risks of missed referrals or unnecessary radiation exposure (~0.1 mSv/scan); (2) families resort to visual self-monitoring, which proves dangerously unreliable (human specificity: 21.7–26.1%; LLM: 0–21.7%); and (3) parental overestimation of deformity-related distress amplifies anxiety [45,46,47,48,57]. LLMs’ catastrophic false positive rates (78–100%) thus carry profound real-world consequences, particularly in resource-constrained settings (surgeon density: 5.5/100,000) where errors divert specialist capacity and exacerbate health inequities [8]. Global extrapolation suggests > 750,000 adolescents could receive false alarms, risking unnecessary anxiety, inappropriate bracing consultations, and avoidable radiation, directly contravening ALARA principles [1,2,3,4,57,58,59].

### 4.4. Architectural Limitations of Transformer-Based LLMs in Scoliosis Assessment

Error analysis identified three primary failure modes inherent to transformer-based LLM architectures when applied to scoliosis evaluation, revealing a fundamental mismatch with biomechanical requirements [60,61,62,63]:

1. Anatomic Misidentification (63% of errors; 78% lumbar-specific):
  - Cause: LLM tokenization decomposes images into discrete units, discarding inherent 3D spatial hierarchies. This reduces complex pelvis–spine biomechanical relationships to statistical co-occurrence patterns rather than structurally constrained anatomical realities.
  - Consequence: Without embedded anatomical priors (unlike CNNs pre-trained on vertebral segmentation), LLMs lack knowledge of spinal ontogenesis (e.g., predictable lumbopelvic tilt relationships). This leads to critical landmark misidentification, such as confusing iliac crests with vertebral endplates.
2. Rotation Blindness (41% under-classification):
  - Cause: Vision Transformers (ViTs) process images as patch sequences, inherently non-geometric representations [41]. This approach reduces spinal torsion to planar noise rather than quantifying rotational vectors orthogonal to the coronal plane. Furthermore, LLMs lack stereoscopic input or shape-from-shading capabilities.
  - Consequence: The inability to infer critical 3D relationships, such as vertebral rotation from rib hump prominence, a pathognomonic feature of scoliosis, results in systematic under-detection of rotational deformities.
3. Threshold Artifacts and Overconfidence:
  - Cause: Autoregressive next-token prediction training incentivizes LLMs to generate plausible diagnostic continuations (e.g., “scoliosis is present”) rather than probabilistically calibrated outputs. This is compounded by biases in web-scale pre-training data, which over-represent severe, conspicuous deformities.
  - Consequence: Minor asymmetries near diagnostic thresholds are catastrophically overinterpreted (e.g., a 9.7° curve misclassified as “14° AIS”), leading to significant false positives.

### 4.5. Mechanistic Convergence and Root Architectural Deficiencies

These failure modes converge and amplify in complex cases, particularly multi-planar deformities, where error severity escalates geometrically with axial rotation (R^2^ = 0.78, *p* < 0.001) [62,64,65]. This convergence stems from core architectural limitations:

1. Spatial Invariance Gap: ViTs lack the convolutional inductive biases (e.g., translation/rotation equivariance) essential for interpreting torsion-dependent deformity progression.
2. Context Collapse: Self-attention mechanisms can inappropriately conflate biomechanically unrelated features (e.g., background clutter with vertebral landmarks), explaining the geometrically increasing errors.
3. Failure to Resolve Biomechanical Interdependencies: Unlike CNNs that hierarchically integrate local features into global structures, transformers treat all image regions uniformly. This prevents differentiation of primary spinal curves from compensatory pelvic adaptations.
4. Absence of Geometric Priors: Crucially, LLMs lack the intrinsic geometric priors found in CNNs trained on rasterstereography [40,43,66]. This fundamental deficit explains their catastrophic proportional bias (slopes ≈ −1.0) when interpreting relationships like rib hump to vertebral rotation, in stark contrast to minimal human bias. The architecture is inherently unable to resolve interdependent spinal parameters.

### 4.6. Path Forward

Specialized CNNs demonstrate superior performance through vertebral rotation integration (CCC > 0.85) [43,67]. Future solutions require

1. Hybrid frameworks incorporating rasterstereography datasets;
2. Uncertainty quantification for borderline curves (8–12°);
3. Region-specific CNNs for pelvis–spine differentiation.

Clinical deployment mandates the following non-negotiable benchmarks: specificity >90%, Cobb concordance ±5°, and LoA ≤ ±10°. Regulatory validation under EU AI Act Article 5 is essential, with “sandbox” testing and real-world monitoring [18,19]. Anthropometric insights (height as deformity predictor; BMI correlations) should inform development while recognizing Hassan et al.’s [24] finding of negligible BMI confounding in measurement reliability (β = 0.12, *p* = 0.38) [68,69]. Crucially, populations most reliant on accessible tools, particularly underserved regions, remain most vulnerable to harm, demanding stringent validation aligned with WHO digital ethics guidelines [70].

Our data reveal an underappreciated dichotomy: disciplines excelling in dynamic screening (e.g., physical therapists using Adam’s forward bend test) may derive limited benefit from static photographic tools. This has implications for AI-assisted screening frameworks. Future work should conduct discipline-specific validation comparing therapists, physicians, and AI using identical photographic datasets; develop hybrid assessment protocols where LLMs quantify visible parameters (e.g., shoulder asymmetry) while therapists retain dynamic examination (e.g., rotational assessment during forward bending); and evaluate therapist–AI symbiosis. Could LLM-generated metrics (e.g., “14° thoracic curve suspected”) combined with therapist-driven rotational analysis improve referral accuracy beyond either modality alone? Such interdisciplinary integration may address the critical sensitivity–specificity trade-off in borderline curves.

General-purpose LLMs demonstrate catastrophic unsuitability for photographic scoliosis assessment, while human evaluation remains diagnostically inadequate. Until validated hybrid systems achieve established benchmarks, photographic interpretation should serve strictly as triage adjunct, never replacing radiographic assessment. Regulatory intervention is urgently needed to prevent technological malpractice in this clinically vulnerable domain.

### 4.7. Limitations and Research Imperatives

While our protocol ensured internal validity, multicenter validation across diverse health-care systems is needed. Future studies must validate 3D-augmented hybrid architectures in high pelvic incidence populations, develop open-access repositories pairing photographs with ISIS2 topography, establish ethical frameworks for AI disclaimers, and quantify psychological impacts of false positives.

General-purpose LLMs exhibit fundamental architectural incompatibility with spinal deformity assessment. Their deployment contravenes regulatory, ethical, and clinical standards. Crucially, clinical photography, while valuable for posture documentation, cannot deliver standalone diagnostic reliability without 3D integration. Future studies should explore adiposity–spinal loading interactions via dual-energy X-ray absorptiometry.

## 5. Conclusions

This prospective validation study conclusively demonstrates that general-purpose multimodal LLMs (ChatGPT-4o and Claude 2) exhibit clinically hazardous inaccuracy in photographic assessment of AIS, evidenced by catastrophic diagnostic failures (0% specificity in ChatGPT; 78.3% false positives in Claude 2), perverse biomechanical reasoning (systematic Cobb angle overestimation of up to +10.74° in thoracic curves; measurement variability of 4.8–7.1× regarding clinical tolerance; inverse concordance [CCC=−0.124] in thoracolumbar curves), and algorithmic malpractice (κ = −0.039 inter-rater reliability) stemming from irreducible architectural limitations: regional analysis confirmed systematic bias patterns, with LLMs demonstrating positive bias in thoracic assessment and negative bias in lumbar/thoracolumbar regions, rotation blindness (ignoring axial torsion in 41% of structural deformities), the inability to translate 2D surface topography into validated 3D spinal kinematics, and pathological overconfidence unsupported by clinical validation. These failures necessitate immediate regulatory safeguards under the EU AI Act (Article 5), including prohibition of LLMs for spinal screening, mandatory disclaimers of unvalidated performance, and centralized adverse event reporting, while future solutions must prioritize hybrid CNN-LLM architectures trained on vertebral rotation datasets with rigorous validation against surface topography standards (e.g., ISIS2) to achieve non-negotiable deployment thresholds (>90% specificity and sub-5° Cobb concordance in multiethnic cohorts); until such criteria are met, photographic AI deployment risks psychological iatrogenesis, unnecessary radiation exposure (~0.1 mSv per false positive referral), and institutionalized malpractice.

## Figures and Tables

**Table 1:** Specialized AI vs. general-purpose AI for spine imaging.

Feature	Specialized Convolutional Neural Networks (CNNs)	General-Purpose Multimodal LLMs
**Design Purpose**	Analyze medical images	Process text, images, and audio broadly
**Validation**	Often validated in specific medical tasks	Limited validation for medical diagnosis
**Accessibility**	Typically proprietary, clinic/hospital-based	Publicly accessible via web/apps
**Example Use**	Measuring Cobb angles from X-rays or surface scans	Answering general health questions

**Table 2:** **Justification for the selection of ChatGPT and Claude in studying AI-assisted scoliosis assessment.** ChatGPT and Claude were prioritized due to their public accessibility and multimodal capabilities, mirroring real-world consumer use for pre-clinical assessment. Their documented propensity for generating confidently unverified medical advice specifically addresses the study’s aim to evaluate high-impact risks in unregulated settings. Alternatives were excluded based on limited public availability, lack of image interfaces, or insufficient ecological validity for family use.

Selection Criterion	ChatGPT and Claude	Excluded Alternatives	Methodological Imperative
**1. Public Accessibility and Real-World Relevance**	Among most widely accessible multimodal LLMs globally Freely available via public web/mobile apps	Proprietary medical LLMs (hospital-integrated) Research-only models (e.g., LLaMA)	Directly aligns with study goal: evaluating tools families actually use for initial pre-clinical assessment
**2. Multimodal Capability**	Native support for image+text input Mirrors real family use (e.g., photo upload + query)	Text-only LLMs (e.g., GPT-3.5) Medical LLMs without public image interfaces	Prerequisite for photographic assessment and real-world interaction paradigm
**3. Representation of “Confidently Unverified Advice” Risk**	Notorious for overconfident medical opinions Reasons: - No medical specialization - Proprietary “black-box” design - Engagement-optimized outputs	Medically fine-tuned models Open-source models (auditable weights/data)	Addresses critical gap: high-risk outputs from high-use tools in unregulated settings
**4. Why Not Other LLMs?**		**Gemini: Regional availability constraints/release timing issues** **Specialized medical LLMs (e.g., Med-PaLM): no public image–input interfaces** **Open-Source MLLMs (e.g., LLaVA): insufficient family adoption**	**Ensures focus on high-impact, consumer-facing tools relevant to study scope**

**Table 3:** Demographic and clinical profiles of the study cohort.

Characteristic	Value	Clinical Relevance
Participants (N)	97	Adequately powered cohort
Age (years)	13.8 ± 2.4	Peak AIS progression age
Female/Male ratio	2.9/1	Matches AIS epidemiology
Height (cm)	157.3 ± 10.8	Tanner stage-appropriate
BMI (kg/m^2^)	19.57 ± 2.98	Consistent with healthy adolescent norms
**Diagnostic Groups**		
AIS (*n* = 74)	76.3%	40 thoracic, 22 lumbar, 12 thoracolumbar
Postural Asymmetry	23.7%	Valid control group

**Table 4:** Diagnostic classification performance of AI models and human evaluators.

Evaluator	Sensitivity (95% CI)	Specificity (95% CI)	PPV (95% CI)	NPV (95% CI)
Claude	82.4% (71.8–90.3)	21.7% (7.5–43.7)	77.2% (66.4–85.8)	27.8% (9.7–53.5)
ChatGPT	**97.3%** (90.6–99.7)	**0.0%** (0.0–14.8)	75.8% (66.5–83.6)	0.0% (0.0–84.2)
Asst. A1	90.5% (81.5–96.1)	26.1% (10.2–48.4)	**79.8%** (69.7–87.7)	46.2% (19.2–74.9)
Asst. A2	90.5% (81.5–96.1)	21.7% (7.5–43.7)	78.8% (68.8–86.8)	41.7% (15.2–72.3)

**Table 5:** Inter-rater agreement metrics for evaluators based on Cohen’s kappa (κ).

Evaluator	κ (95% CI)	Agreement Level	Clinical Interpretation
A1	0.196 (0.012–0.380)	Poor	Barely above chance
A2	0.147 (−0.038–0.332)	Very poor	Questionable reliability
Claude	0.045 (−0.120–0.210)	None	Unacceptable for use
ChatGPT	−0.039 (−0.214–0.136)	Worse than chance	Hazardous systematic error

**Table 6:** Inter-method concordance for regional Cobb angle estimation (Lin’s Concordance Correlation Coefficient, CCC).

Region	Evaluator	CCC (95% CI)	Agreement Level	Clinical Meaning
**Thoracic**	Claude	0.024 (−0.158–0.204)	None	Random fluctuation
	ChatGPT	0.040 (−0.089–0.167)	None	No correlation
	A1	0.331 (0.144–0.496)	Poor	Minimally acceptable
	A2	0.350 (0.212–0.547)	Poor	Best human performance
**Lumbar**	Claude	0.160 (0.032–0.337)	None	Unreliable
	ChatGPT	0.0004 (−0.183–0.184)	None	Complete failure
	A1	−0.080	Discordant	Inversion tendency
	A2	−0.090	Discordant	Hazardous bias
**Thoracolumbar**	Claude	0.030 (−0.249–0.042)	None	Near-zero agreement
	ChatGPT	0.000 (−0.299–0.059)	None	Worst overall

**Table 7:** Global measurement agreement and clinical risk based on Bland–Altman analysis.

Evaluator	Bias (°)	LoA Range (°)	95% LoA (°)	Clinical Risk
Claude	+4.96	**71.08**	−30.58 to +40.50	Unacceptable
ChatGPT	+10.74	64.37	−21.45 to +42.92	Critical overestimation
Asst. A1	+1.51	63.04	−30.01 to +33.03	Moderate risk
Asst. A2	+0.76	58.25	−28.37 to +29.88	Least error

**Table 8:** Concordance Correlation Coefficients (CCCs) across curve subtypes.

Curve Type	Best Evaluator	CCC	Worst Evaluator	CCC
Thoracic	A2	0.350	ChatGPT	0.040
Double Major	A1	0.287	Claude	−0.112
Thoracolumbar	Gold Standard	N/A	ChatGPT	−0.100

## Data Availability

The data presented in this study are available upon request from the authors. The data are not publicly available due to ethical restrictions.

## Abbreviations

The following abbreviations are used in this manuscript:

LLMs	Multimodal Large Language Models
AI	Artificial Intelligence
AIS	Adolescent Idiopathic Scoliosis
CNN	Convolutional Neural Network
ST	Surface Topography
TAPS	Trunk Appearance Perception Scores
GDPR	EU General Data Protection Regulation
CCC	Concordance Correlation Coefficient
LoA	Limits of Agreement
ALARA	As Low As Reasonably Achievable
API	Application Programming Interface
